# Matrix community models for ecology and evolution

**DOI:** 10.1038/s44185-023-00031-5

**Published:** 2023-12-04

**Authors:** David A. Lytle, Jonathan D. Tonkin

**Affiliations:** 1 Department of Integrative Biology, Oregon State University, Corvallis, OR 97331 USA; 2 School of Biological Sciences, University of Canterbury, Private Bag 4800, Christchurch, 8140 New Zealand; 3 Te Pūnaha Matatini Centre of Research Excellence, University of Canterbury, Christchurch, New Zealand; 4 Bioprotection Aotearoa Centre of Research Excellence, University of Canterbury, Christchurch, New Zealand

**Keywords:** Community ecology, Ecological modelling, Population dynamics

## Abstract

Ecological communities are shaped by biotic interactions as well as environmental forces, and both must be incorporated to obtain models capable of forecasting realistic community dynamics. Many community models first specify pairwise biotic interactions and then secondarily examine how extrinsic factors such as abiotic conditions affect species abundances. A disadvantage of this approach is that the species interactions themselves are often environment and context specific, making parameterization difficult. We propose an alternative approach, matrix community models (MCMs), which are sets of matrix population models linked by an assumption of aggregate density dependence. MCMs incorporate detailed species autecology but are neutral with respect to pairwise species interactions, instead allowing interactions to be revealed within the model structure. These model-revealed species interactions, including competitive exclusion, facilitation, and interference competition, shape the distribution and abundance of species within communities and generate empirically testable predictions about species interactions. We develop a framework for building MCMs using vital rates in a stochastic, multispecies framework. Single-species matrix population models are connected via an assumption of aggregate density dependence, pairwise species interactions are estimated with sensitivity analysis, and community trajectories are analyzed under different environmental regimes using standard statistical tools and network analysis. MCMs have the advantage that pairwise species interactions need not be specified a priori, and that mechanistic demographic-environment linkages permit forecasting of community dynamics under novel, non-stationary environmental regimes. A challenge is that species’ autecological vital rates, such as fecundity, growth and survivorship, must be measured under a diverse range of environmental conditions to parameterize the models. We illustrate the approach with examples and discuss prospects for future theoretical and empirical developments.

## Introduction

Species interactions occupy a central place in understanding the distribution and abundance of species within ecological communities. For example, in Lotka-Volterra models^1,2^ and models derived from this starting point, species are defined largely by the way they interact with other species, via coefficients that represent predation rates, competition, or mutualistic relationships. This framework has yielded a rich body of ecological theory that has been used to understand pairwise species interactions, assembly and disassembly of communities, and the dynamics of entire ecosystems. The study of species interactions has been one of the cornerstones of modern ecological theory from the 20th century forward^3–5^.

In practice, however, pairwise species interactions can be challenging to quantify in both natural and laboratory settings. While some well-known species interactions are strong (as measured by interaction strength, the per-capita effect of one species on another), most pairwise species interactions are in fact weak^6^. The prevalence of weak interactions has been attributed variously to omnivory, ontogenetic shifts in diet, and frequency-related shifts in prey preference, among other mechanisms^7,8^. Analysis of full communities is further highlighting the importance of community context (where higher order interactions alter community dynamics), environmental variability (effect of disturbance events or different year-types), and seasonality (cyclical changes in food, temperature, and other environmental factors) in altering the strength and even the direction of pairwise species interactions^9–12^. Indeed, the effects of pairwise interactions may fade or disappear entirely in the context of complex multispecies communities^13,14^.

For ecologists striving to understand the distribution and abundance of species and model complex communities, the context-dependence of species interactions presents a conundrum – if the fundamental variates in models are refractory or difficult to quantify, how can we accurately forecast community dynamics into the future? As climate change and other anthropogenic activities produce novel environments and community arrangements^15–17^, how can we specify the correct values for the species interaction coefficients these models depend on? Demographic models provide a framework for understanding the distribution and abundance of species under changing environmental conditions. While traditionally applied to single species, demographic approaches such as matrix population models can be applied to understanding whole communities. An advantage to demographic models is that they permit a direct connection between species’ vital rates – parameters related to reproduction and survival – and population trajectories across a wide range of environmental conditions. As we will show below, if we first begin with well-defined demographic models that capture how co-occurring species fluctuate in variable environments in a community-wide density-dependent framework, we can estimate species abundances and then infer how species interactions arise as an emergent property of the system.

### Species interactions are an outcome – interaction neutral models

**Fig. 1:**
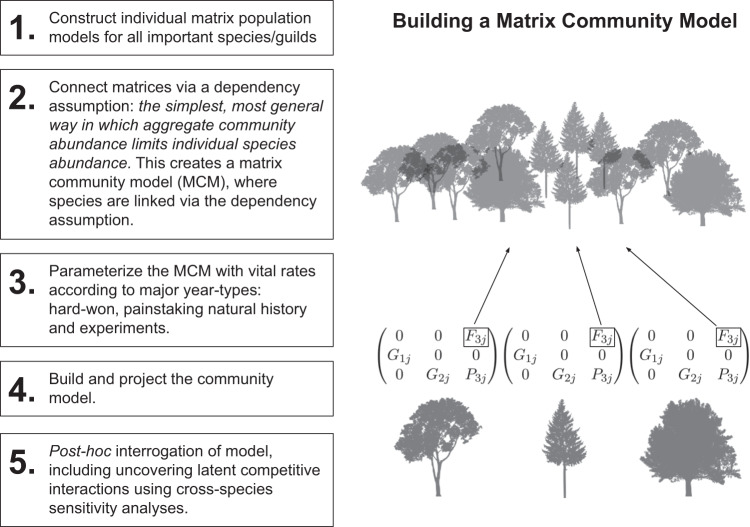
Building and projecting a matrix community model. The right panel depicts that fecundity, in this case, is a function of aggregate density dependence. In models of riparian vegetation, the assumption that there is a finite amount of open space available for seedling recruitment serves as the dependency assumption.

If pairwise species interactions are so fickle, then why not leave them out of community models entirely, or at least omit them from initial model parameterization? This is the “interaction neutral” perspective, where species interactions are omitted from model structure and then inferred later from model output. In model building, our first and most important order of business is to carefully parameterize how individual species interact with their abiotic environments, under a variety of environmental conditions, in terms of growth, survivorship, fecundity, and other relevant vital rates (Fig. 1). Species are then linked together by a general assumption of aggregate density dependence which allows them to compete for a common resource, such as space or total resources available to all species combined. Species interactions are latent in the model, because even though species compete implicitly for some finite resource, pairwise species interactions – such as competitive exclusion, facilitation, and interference competition – are not specified a priori in the model structure. If they are of interest, species interactions are instead estimated post hoc from sensitivity analysis of the model itself, producing estimates of interaction strengths under a variety of environmental regimes and community contexts. In this sense, species interactions are treated as an epiphenomenon of species living together in a finite world that experiences a constantly changing set of environmental conditions. Species interactions are an outcome of these processes, not a characteristic biological attribute of the organisms^18^.

### Matrix community models

Matrix community models (MCMs) provide a useful demographic framework for understanding the population dynamics and abundance of species under changing environmental conditions. Matrix community models are sets of single-species matrix population models that are linked together to capture community-wide dynamics. In the treatment below, we develop an approach that deliberately omits the strength and direction of pairwise biotic interactions from the general model structure and focuses instead on the vital rates of individual species as they relate to different environments. Biotic interactions are then estimated post hoc from model sensitivity analysis. Vital rates include survivorship, fecundity, and stage- or age-specific transition probabilities, each as a function of different environmental types. In matrix population model notation, each species *j* in the community is specified as an age- or stage-classified matrix (*i* stages) with its own set of vital rates in matrix model form

1 $${{\bf{n}}}_{j}\left(t+1\right)={{\bf{A}}}_{j}\left(t\right){{\bf{n}}}_{j}\left(t\right)$$

where **n**_*j*_(*t*) is a vector containing age or stage abundances and **A***j*(*t*) is a set of transition matrices containing vital rates that fluctuate over discrete time intervals *t*. This can be accomplished by drawing sets of vital rates from a set of environment types, or using integral projection to connect a continuum of vital rates to environmental states^19^. For a species that lives about three years and starts reproducing in the third year,

2 $${{\bf{A}}}_{j}\left(t\right)=\left(\begin{array}{ccc}0 & 0 & {F}_{3j}\\ {G}_{1j} & 0 & 0\\ 0 & {G}_{2j} & {P}_{3j}\end{array}\right)$$

where *F*_*ij*_ denotes fecundity, *G*_*ij*_ is cross-stage transition probability, and *P*_*ij*_ is probability of remaining in a particular stage. Because vital rates in Eq. 2 fluctuate according to different environmental conditions (wet vs. dry years, or disturbances of varying magnitude) there may be any number of **A***j*(*t*) that correspond to each environmental type, and the model becomes time-varying or stochastic if these year types occur at random. The theory of structured populations in stochastic environments is well developed and includes methods for understanding both asymptotic and transient dynamics of individual populations, estimation of population growth rates, and sensitivity analysis^20–22^.

### Deriving species interactions from matrix community models

Species exist in communities, so individual species matrices must be linked together in some way to understand how they interact. This can be achieved by introducing a dependency assumption, where density dependence acting on any one species becomes a function of the abundances of other species in the community. This community model is neutral with respect species interactions; pairwise interactions are not specified in the model structure, but they are inferred post-hoc from analysis of the model output. The coupling of individual species matrices into communities opens up new challenges and possibilities. Although MCMs have been explored analytically, efforts have involved small numbers of interacting species in non-stochastic environments^23–26^. Adding larger numbers of species to the community, especially in the context of stochastic environments, introduces challenges that push model analysis out of the grasp of analytical solutions and into the realm of simulations. As such, thorough analysis of MCMs has only become possible with the availability of greater computational power.

**Fig. 2:**
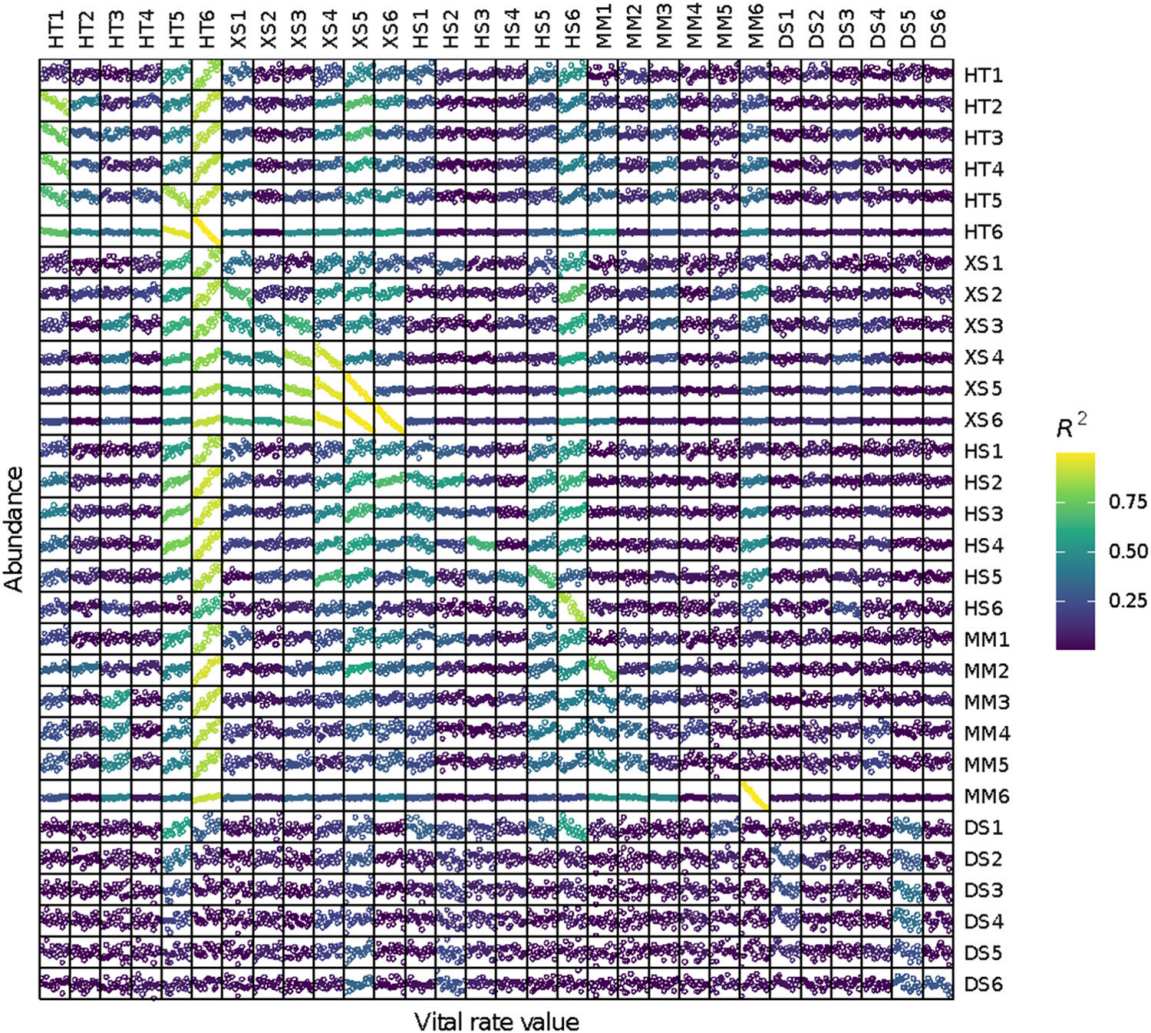
Latent interactions derived from cross-guild/stage sensitivity analysis (guilds were used in this specific study for model transferability). The relevant vital rate (here, mortality due to flood events; scaled from 0 to 1) was changed by increments of 0.01 in the vicinity of its actual value and population size was recorded. At each increment, this was repeated independently up to 1000 times to achieve stable convergence on mean values. Sensitivity was calculated as the slope of the average population size of a guild regressed against the vital rate, which is analogous to taking the partial derivative of population size with respect to the vital rate of interest (Lytle & Merritt 2004). Only sensitivities with R^2^ > 0.3 were retained for network analysis. Because each guild contains six stages, the matrix depicts both within-guild and among-guild effects. The diagonal depicts self-effects, where an increase in mortality causes a decrease in abundance of that guild/stage. Positive relationships suggest competition, where an increase in mortality rate in one guild/stage allows another one to increase. HT, XS, HS, MM, and DS represent riparian plant guilds that possess similar vital rates (respectively, hydroriparian tree, xerophytic shrub, hydroriparian shrub, mesic meadow, and desert shrub) and numbers indicate stage class.

**Fig. 3:**
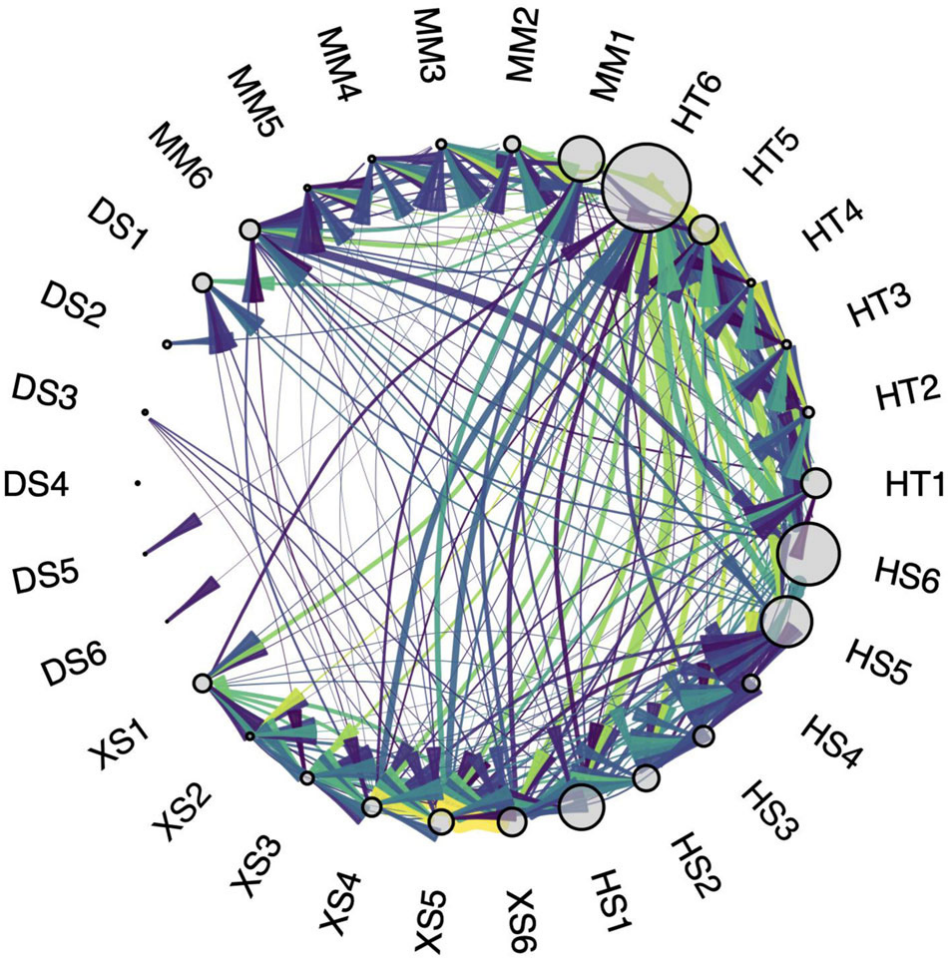
Network analysis of riparian vegetation showing how changes in a vital rate in one guild/stage affects other members of the community. Edge thickness denotes the slope of the relationship and edge color denotes R^2^ (colors and node labels as in Fig. 2). Note that a relationship can have a strong fit (high R^2^) but low magnitude of effect on abundance (shallow slope). The size of each node on the graph depicts the average abundance of that guild/stage. In this riparian community, which was modeled under a natural flow regime of flood and drought frequencies, mature hydroriparian trees (HT6) are a keystone of the ecosystem because of their high connectance to other nodes. In western U.S. riparian areas, HT6 often consists of cottonwood (*Populus fremontii*) gallery forest.

Below we develop a framework for building and analyzing matrix community models (Fig. 1) by quantifying species interactions via sensitivity analysis (Fig. 2) and exploring novel community trajectories using networks (Fig. 3). We illustrate the approach with recent empirical examples exploring communities of riparian vegetation^27^ and fish^28^, and discuss prospects for new theoretical and empirical explorations.

### Building a matrix community model

#### Construct individual matrix population models

Matrix population models are a cornerstone of ecology, evolution, and conservation biology^29,30^. The methods are diverse and can accommodate variability in age or stage structure, environmental setting, and timescale of interest. Matrix population models are also mechanistic in that they incorporate parameters and processes with direct population-biological meaning, such as age-specific survivorships, fecundity, and age or stage transition probabilities. The construction of single-species matrix population models is well-covered elsewhere and thus beyond the scope of this paper, but some general tips apply. It is advantageous, though not strictly required, to choose a timescale common to all species in the community, such as annual time steps. This allows all species to share a common model structure, easing the implementation of multi-species analysis. The next choice is which species to include. Ideally one would include all species in the community, even “unimportant” rare species that may be observed in low abundances in extant communities, since these could become more numerous under certain environmental regimes. Including all species is not always practical since vital rate information may be lacking for some taxa. One remedy is to group species into guilds that possess similar life histories and environmental responses^31^. Although guilds may simplify analysis in some cases, it is not necessary to use them, especially when vital rates are available for individual species. Because matrix community models include density dependence, it is important to include taxa that are significant in terms of numbers and biomass, at least under some environmental conditions, since omission of a key species could affect the relative abundance of other members of the community.

#### Specify the dependency assumption

Although species are initially modeled as independent autecological units that interact only with their environments, the population dynamics of individual species must be linked together in some way to form communities. This can be accomplished via a dependency assumption, which specifies how density dependence acts across the entire community in aggregate. The dependency assumption is the simplest, most general way in which aggregate community abundance limits individual species population growth rates. One or more of the life history parameters for each species will approach zero as the entire community approaches this limit, which is essentially a carrying capacity, *K*^32^. If density dependence acts on fecundity, for example, the reproduction of any species *F*_*j*_ becomes a function of the summed quantity *n*_*S*_ (measured as number of individuals, biomass, or any other abundance metric) of all *S* species of all stages in the community, or *F*_*j*_(*n*_*1*_*, n*_*2*_*, n*_*3*_*,…,n*_*S*_). As the sum total of *n*_*S*_ approaches *K*, reproduction will approach zero for all species. For models of riparian vegetation, the assumption that there is a finite amount of open space available for seedling recruitment has previously provided a reasonable dependency assumption (Eq. (1) in ref. ^27^). At each annual timestep, the space occupied by each stage of each species was summed and subtracted from the total available riparian area, and this quantity of available space became an upper limit on recruitment for each species. The total amount of available habitat, *K*, was estimated from aerial photos and study plots. In this way the population dynamics of each species in the community can have an effect on the recruitment of any other species. This approach is fairly straightforward for communities such as riparian plants which occupy a single trophic level and thus only experience competition and facilitation, but what about multi-trophic animal communities? Rogosch et al. (ref. ^29^) chose a dependency assumption for fish communities where the egg survivorship of each species was a function of the total fish biomass of all species present in the river (their Eq. (2)). The upper bound on total fish biomass, *K*, was calculated as the average fish biomass observed within a study reach over a long time series. Even though the model included species that are known to be predators on other species in the community, Rogosch et al. (ref. ^29^) chose not to include these known species interactions in the model structure. Rather, the approach taken was to explore how well the model could predict empirical fish abundances by considering only the autecological interactions of species with different year-types under a generalized dependency assumption of finite fish biomass.

#### Measure the vital rates

This is the hard work. Measuring vital rates can involve remeasuring individuals repeatedly over many years, collecting data under a variety of physical settings and year types, analyzing chronosequences of aerial photos of forest plots, or conducting experiments under carefully-controlled conditions. This includes collecting vital rates that pertain to relevant situations, such as mortality of riparian trees during floods or droughts (a different set of vital rates for each circumstance) or mortality rates of fish under different year types that differ in flood timing and magnitude. Although it is challenging to obtain detailed vital rate data for many species in a community, the effort is cumulative and vital rate data are becoming available for a growing number of plant and animal species^33,34^. We note also that there is a whole suite of hybrid modeling approaches becoming available to estimate vital rates from diverse data structures, including borrowing strength from other species and fusing data^35^.

#### Build and project the community model

The vital rates and the dependency assumption need to be combined into a single modeling framework. Previous efforts discussed here used coupled matrix population models, where vital rates for individual species or guilds are included in a Leslie or Lefkovitch matrix, and the dependency assumption is included as some form of aggregate density dependence as described above. While convenient, a matrix population model structure is not strictly required. In principle, any population model structure that allows for multiple year types and density dependence of species, such as logistic growth models^36^, could be aggregated into a multispecies model. This is a topic in need of further exploration.

Because a full community model incorporates vital rates conditional on specific year types, the way in which the model is projected forward in time will affect the model results. One approach is to assume that year types occur at random with a given frequency, resulting in a stochastic model structure that meets assumptions of independent, identically-distributed environmental states^37^. The way in which models are projected forward in time is quite flexible, however, and can allow the exploration of any number of what-if scenarios. One can explore changes in the frequency of year types^38^, autocorrelation among year types^39^, or specified sequences of year types that match historical time series^28^. This may be one of the chief advantages of using a population-dynamic model framework such as the one described here instead of a statistical analysis – the modeling framework can accommodate a great variety of possible environmental scenarios, including those imposed by extreme climate shifts and non-stationary changes to climate regimes^17^.

The particulars of the community model structure will also affect the long-term dynamics of the model. In a riparian vegetation community model^27^, it was assumed that a single tree could produce enough seed to populate all available open space along the modeled river section and that seedling recruitment was independent of adult tree population size. This led to a rescue effect where the occurrence of a single favorable year could return a small population back to a higher abundance, so long as the other conditions governing recruitment were met. In a fish community model^28^, by contrast, the number of eggs produced was a function of the number of reproductive adults. This type of fecundity can lead to stochastic dynamics where low numbers of individuals can quicken the extirpation of a species. The models discussed above examine a single, finite patch of habitat. Placing the habitat in a metacommunity context could produce novel model dynamics and results, and deserves further attention.

#### Identify latent competitive interactions using cross-species sensitivity analysis and network tools

A sensitivity analysis measures the effect that changing a single vital rate has on population growth rate^40^ or population size^41,42^. In traditional single species population models, sensitivities measure the population-level effect of perturbations to vital rates in a single stage. In a multispecies model, sensitivities measure how perturbation to a vital rate affects abundance of a focal species (and its various ages or stages) as well as other species in the community. Cross-species sensitivity analysis produces an entire matrix of pairwise interactions that reveal how vital rate changes in any one species or stage could affect any other species (Fig. 2). A cross-species sensitivity matrix produced in this way is analogous to a net effects matrix derived from systems of Lotka-Volterra equations, which encapsulates pairwise effects on species abundance after accounting for all direct and indirect effects^18^. In essence, these relationships represent a type of compensatory dynamic, whereby reductions in one species or stage–as a result of altering a vital rate–creates opportunities for others^43^. The cross-species sensitivity matrix is conditional on one particular environmental regime; changing the environmental regime can result in an entirely new cross-species sensitivity matrix, sometimes with major differences in which species have the largest community-wide effects^38^. Because MCMs are nonlinear due to density dependence, numerical simulations must be used to calculate sensitivities. Obtaining sensitivity estimates may be the most computationally expensive part of this modeling process.

The cross-species sensitivity matrix is rich with information about how species fluctuate in population size and interact with each other under a specific environmental regime. Digesting this information so that it is usable can be accomplished using the tools of network theory, which examine the strength and number of linkages among species (Fig. 3). Each node within the network represents a species or guild, or an individual stage of a species or guild. Positive and negative net effects are represented by the edges (links) that connect nodes. It is necessary to define what constitutes a significant interaction. For example, Tonkin et al. (ref. ^38^) defined significant interactions as those sensitivities with an R^2^ value exceeding 0.30 and a slope significant at *p* < 0.01.

It is important to understand what sensitivity networks do and do not represent. An edge between two nodes is a measure of how a change in a specific vital rate in one species-stage will change relative abundances in another species-stage. It is tempting to interpret edges as the per-capita effect of one species on another, as is the case with a traditional interaction matrix. In the context of matrix community models, the interpretation is analogous but differs in that the interactions are mediated by changes to a specific vital rate rather than changes to abundances per se. Nevertheless, the effects of changing vital rates of species *i* on species *j* are mediated by altered abundances in species *i*. Thus, it is theoretically possible to examine this relationship more directly in line with a traditional community matrix approach^18^.

In complex communities, many interactions are possible and it is difficult to visualize community dynamics. Network analysis is a useful way to summarize these effects and summarize how they change across environmental regimes^44^. There are a wide range of potential network-level metrics available to characterize such networks, including network connectance, or how connected the network is via links among nodes; reciprocity, or how often links between two nodes are bi-directional; link density, or the average number of links per node; modularity, or how compartmentalized the interactions are in the network; and the relative frequency of different motifs (small, repeatable network subgraphs). Similarly, there is a wealth of potential node-level metrics available to characterize the role of species or species-stages in complex networks, including various forms of degree, or how connected a node is to other nodes, and centrality, or how often links pass through a node. In the case of MCMs, analyses for directed unipartite networks are most appropriate^44^. However, incorporating multiple trophic levels or interaction types (bipartite or multipartite networks) is not beyond the realm of possibility with further developments of the MCM approach.

## Discussion

On the one hand we have neutral biodiversity community models that require little information about individual species’ biology^45,46^. On the other hand we have food web and community models that lean heavily on biotic interactions in model parameterization^47,48^. In MCMs, species are well-described by vital rates such as environment-specific reproductive and survival rates derived from empirical data, but pairwise biotic interactions are not specified up front. Thus, MCMs represent a middle ground relative to these approaches. Treating species in this way allows us to go beyond broad statements about biodiversity patterns and begin to understand how the abundance and distribution of particular species might change under future environmental scenarios as a result of environment-vital rate interactions in a multispecies density dependent setting. The model structure of MCMs also allows us to generate testable predictions about how species interact with each other, and how these interactions might vary under different environmental circumstances.

Vital rates can be difficult to obtain because MCMs requires the observation of organisms across a large range of environmental conditions. However, vital rates reveal much about individual species and community dynamics. A model is useful when it leverages the information present in empirical data to give the most explanatory power under the greatest variety of circumstances. Vital rates contain implicit information about how species interact with each other and with the environment, although this information may not be readily apparent when studying species in isolation. For example, the fact that an oyster produces millions of gametes per spawning event (high fecundity value) immediately suggests that per-gamete survivorship is probably low. We know intuitively that there is a tradeoff between the number and quality of offspring because the energy that an organism can allocate to reproduction is finite^49^. But sometimes the information hidden in vital rates is only apparent in the context of complex communities, and requires a particular model structure to reveal it. For example, cottonwoods are often the dominant riparian tree in free-flowing rivers in western North America. This occurs because cottonwoods possess a combination of vital rates that confer success under river flow regimes that experience occasional major floods that facilitate establishment of new seedlings^50^. However, a shift to a homogenized flow regime with fewer flood events and more frequent droughts favors the establishment of non-native tamarisk, which possesses a life history similar to cottonwood but is more drought tolerant^51^. Exploring these vital rates in the context of MCMs reveals these differences and allows community level predictions of species’ relative abundance on the landscape^27^. In this way, MCMs unpack the information hidden in vital rates and allow us to observe, and predict, community-level differences that arise from differences in species autecologies.

One could argue that our lack of information about individual species’ vital rates leaves us in the same position as our lack of knowledge of pairwise species interaction terms – that we will be forced to populate our models with educated guesses rather than empirically-measured estimates. There are several reasons why this is likely not the case. Because vital rates represent a fundamental mapping between an organism and its environment, they may be less variable in different ecological contexts than species interactions. A vital rate, such as an age-specific survivorship for a specific year-type, is the observed result of one species experiencing one specific environmental type. These data can be obtained from cross-sectional studies along environmental gradients, observations of marked populations through time, or from controlled experiments. As the autecological library of individual species grows, from observations obtained across a greater variety of environments, this information can be readily incorporated into models. This is not necessarily the case with species interaction coefficients, which are expected to change in the context of other species in the community^7,52^. Vital rates also represent simpler, more fundamental quantities than species interactions. An interaction coefficient is a function of two (or more) species interacting with each other in addition to interacting separately with the environment. As such, species interaction coefficients are by definition higher-order phenomena, and thus potentially more context-dependent; the number of contexts is potentially infinite. While the task of compiling autecological data for a large number of species may be daunting, it is a definable task that is ultimately achievable. Parameterizing MCMs shifts our focus from measuring species interactions to measuring vital rates, a research agenda that restores descriptive, autecological natural history to its former prominence^53^.

Matrix community models are strongly driven by differences in the magnitude and frequency of disturbances or different year types. While sometimes viewed as “noise” that needs to be explained away with caveats, studies are beginning to embrace environmental variation as a driving force in understanding ecological interactions^54^ and evolutionary trajectories^55^. MCMs include environmental variation in the fundamental model structure, and some of the most striking findings arising from MCMs concern how community structure, and the ecological importance of particular species, can change as environmental regimes change^38^. The interaction between environmental variation and species persistence has many analogies to the lottery model of Chesson & Warner (ref. ^56^), a connection that deserves further attention.

In addition to MCMs, other community modeling approaches can be useful for estimating species interactions from other sources of data, but require great care in interpreting or assigning ‘interactions’. For instance, multivariate autoregressive models (including state-space variants; MARSS) allow estimation of interaction strengths from time series data in addition to a species-environment covariance matrix^57,58^. Joint Species Distribution Models (jSDMs) offer the ability to infer species interactions from data on species co-occurrence, although missing environmental predictors may impose limits^59,60^. MCMs differ from these approaches in that they employ a mechanistic population biology structure, rather than relying on statistical inference to identify interactions. Because they are mechanistic, MCMs may be better able to project population and community structure into future, nonstationary environmental regimes that are beyond the predictive envelope of statistical methods^17^, but answering this will depend on comparing MCMs to the various modeling approaches. MCMs may also help resolve debates over whether species interactions can be inferred from co-occurrence data at all^61^.

### Future challenges for matrix community models

#### Cross-species sensitivity analysis and species interactions

Ideally, community models should be useful for both forecasting population dynamics under specific scenarios and revealing generalities about how biological communities operate. Cross-species sensitivity values derived from MCMs are hypotheses about how species might interact under particular environmental regimes. This is a potentially rich source of information that can be used to understand mechanistically how communities work, and also make predictions about species interactions that can be empirically tested. This hypothesis-generating ability is a key, often unexplored, benefit of ecological forecasts for the development of ecological theory. Which species interactions are expected to be strong under novel environmental regimes? Are there some species interactions that persist across a wide variety of environmental regimes? Can we use these results to identify general rules that govern how changes in abundance of one species affects another as environments change?

#### Evolution and eco-evo feedbacks in a full community context

In single-species matrix models, sensitivities describe how changing a vital rate would change population growth rate or some other model parameter of interest. Sensitivities are also estimates of selection gradients, because natural selection will favor values of vital rates that increase population growth rates^29^. It seems intuitive, then, that cross-species sensitivity analyses derived from MCMs should tell us something about how species could evolve in a full community context. A vital rate (such as mortality, growth rate, or fecundity) is not an organismal trait that can be directly changed by natural selection, however, so a link would need to be made between the vital rates used in a model and particular features of the organism that can evolve (body size, allocation to growth vs. reproduction, resource utilization, etc.). Another challenge is that evolutionary change in one species will have rippling effects throughout the community^62,63^, necessitating the continuous recalculation of model outputs as the system evolves^9^. This presents computational challenges – as one (or more) species evolves, other species in the community may evolve in response to it, creating the potential for strongly nonlinear dynamics in model behavior. Advances are being made that incorporate eco-evo feedbacks into matrix population models^64^, and perhaps these insights can be eventually be integrated into MCMs.

#### A call for more theory and more analysis tools

The matrix community models discussed here are stochastic, non-linear, multispecies models that incorporate density dependence. Depending on the specifics, model analysis may also require analysis of transient dynamics rather than analysis at equilibrium conditions^64^. Because of their mathematical form, MCMs containing more than two species have thus far been analyzed using simulation approaches, which limits the discovery of general model principles and behaviors. Can we identify general principles or patterns that hold true under a variety of conditions? For example, do species with characteristic sets of traits respond predictably to certain environmental regimes? How do different forms of density dependence affect model output? What is the effect on model behavior of adding more species (or removing species)? All of these are important questions, but it remains to be seen if they can be answered analytically, with simulations, or with some combination of the two approaches. Developing a more generalized model structure that can accomodate a wide variety of applications is also an area in need of development. Current MCMs are tailored to specific systems, such as riparian forests and riverine fish communities, and the model structures reflect the particulars of these organisms and ecosystems. A more generalized implementation, with corresponding code, would facilitate exploration of general model behavior as well as specific applications in other ecosystems.

#### Metacommunity context

In the matrix community model developed by Rogosch et al. (ref. ^28^), if the model is projected far enough into the future all fish species eventually go extinct except one. The surviving species is typically the one with the highest single-species stochastic rate of population increase under the given environmental regime (sequence of flood and drought year-types in this case). At first glance this appears to be a failure of the matrix community modeling approach – that the model fails to allow the stable coexistence of species in communities. This perspective assumes that there must be some mechanism (storage effect, facilitation, etc.) that allows species to coexist in communities through time. An alternative view is that we should expect the local extirpation of most species when space and population sizes are finite, especially in stochastic environments. In the absence of spatial rescue effects, if we throw seven fish species into an isolated 1-km reach of river and return decades later, it would be unsurprising to see only a single species remaining, if any remained at all, due to chance demographic events that occur in variable environments. Indeed, it is a general result of stochastic population models that finite populations eventually go extinct in fluctuating environments, due to geometric averaging of population growth rates over time^65,66^.

There are two potential ways of exploring this result. First, the analysis of transient dynamics can be used to explore population and community trajectories at meaningful timescales^42,67^. Recognition that communities may never achieve equilibrium is also leading to the development of new methods for understanding non-equilibrium community tendencies^68^. Second, a metapopulation model approach^69^ would allow species to recolonize patches of habitat, and also allow habitat patches to experience environmental states at different frequencies and synchronies. Single-species metapopulations could also be aggregated into metacommunities^70^ within an MCM framework, as has been done for other community models^71^.

#### Can we obtain unbiased vital rates?

The argument can be made that vital rates, just like biotic interactions, are measured in the context of other species and environmental states, and are thus biased in some way. For example, life history traits of many organisms vary across latitudinal and elevational gradients, suggesting that these traits change in response to context^72,73^. The variability of species traits and vital rates across populations and ecosystems is an open question and an active area of research^74–77^. MCMs assume that variation in species’ vital rates is lower than variation in pairwise competition coefficients when looking across a diversity of environmental regimes or community contexts. This is an assumption that can be directly tested with empirical data.

### Summary

Matrix community models flip the narrative on species interactions by omitting them entirely from the model structure. Instead, MCMs rely on autecological processes to show us how species interactions arise from species living, dying, and reproducing in variable environments. Under this view, pairwise species interactions are not a tangible force that can be measured and then entered as a parameter value, but rather an outcome of more fundamental interactions between organisms and the dynamic, fluctuating environments in which they live. This is not a new view of ecological communities; debate over the importance of biotic vs. abiotic drivers is a recurring theme in community ecology. Clearly, species change in abundance due to environmental conditions in addition to direct and indirect competition with each other. The question is, if we want to forecast the abundance and diversity of organisms in future environments altered by shifting climates and anthropogenic change, which features of the organisms do we prioritize for inclusion in our models? MCMs provide an opportunity to elevate species-environment relationships in a way that simultaneously captures the most important way in which species interact with each other.

## Data Availability

Data and code archives Model code and data for analyses discussed herein are archived in figshare and zenodo: figshare 10.6084/m9.figshare.4652608 zenodo 10.5281/zenodo.1309024.
